# The Influence of Domestic Overload on the Association between Job Strain and Ambulatory Blood Pressure among Female Nursing Workers

**DOI:** 10.3390/ijerph10126397

**Published:** 2013-11-27

**Authors:** Luciana Fernandes Portela, Lucia Rotenberg, Ana Luiza Pereira Almeida, Paul Landsbergis, Rosane Harter Griep

**Affiliations:** 1 Laboratory of Health and Environment Education, Oswaldo Cruz Institute, Avenida Brasil, 4365, Manguinhos, Rio de Janeiro, 21040-360, Brazil; E-Mails: rotenber@ioc.fiocruz.br (L.R.); analu05@gmail.com (A.L.P.A.); rohgriep@ioc.fiocruz.br (R.H.G.);; 2 School of Public Health, Downstate Medical Center, State University of New York, 450 Clarkson Avenue, Brooklyn, NY 11203, USA; E-Mail: paul.landsbergis@downstate.edu

**Keywords:** blood pressure, ambulatory, job strain, women, work, gender, health

## Abstract

Evidence suggests that the workplace plays an important etiologic role in blood pressure (BP) alterations. Associations in female samples are controversial, and the domestic environment is hypothesized to be an important factor in this relationship. This study assessed the association between job strain and BP within a sample of female nursing workers, considering the potential role of domestic overload. A cross-sectional study was conducted in a group of 175 daytime workers who wore an ambulatory BP monitor for 24 h during a working day. Mean systolic and diastolic BP were calculated. Job strain was evaluated using the Demand-Control Model. Domestic overload was based on the level of responsibility in relation to four household tasks and on the number of beneficiaries. After adjustments no significant association between high job strain and BP was detected. Stratified analyses revealed that women exposed to both domestic overload and high job strain had higher systolic BP at home. These results indicate a possible interaction between domestic overload and job strain on BP levels and revealed the importance of domestic work, which is rarely considered in studies of female workers.

## 1. Introduction

A significant number of studies have documented the association between exposure to a stressful psychosocial environment at work and cardiovascular diseases [1]. Special attention has been paid to cardiovascular risk factors (hypertension, obesity, smoking habits, metabolic syndrome) and their relation with occupational stress. The increase in the incidence of hypertension worldwide [2] parallels transformations in the nature of work due to economic globalization, including increases in work stressors [3]. Thus, occupational health research emphasizes the importance of psychosocial factors related to work organization as potential risk factors for ill health. 

The job strain model represented a breakthrough in psychosocial work environment research. A number of studies have indicated that the psychosocial conditions of the work environment, especially the combination of high psychological demands and low decision latitude (job strain), associated with an increased risk of cardiovascular diseases [4]. There is strong evidence that job strain plays an important role as a risk factor for hypertension [1,5,6]. 

Despite the substantial increase in women participating in the labor market in recent decades, most publications on occupational health deal with men [7]. This situation also applies to studies that specifically investigate the effects of job stress on blood pressure [3]. 

Besides, a meta-analysis of ambulatory blood pressure studies revealed that associations of job strain with blood pressure elevation are stronger for men than for women [8]. One should also consider that data among women are less consistent that data on men [3], so that the original concept of job strain was proposed by some authors to be more applicable to men than to women [9]. For instance, in a cohort study of workers engaged in several jobs, Ohlin *et al.* [5] found that job strain predicts a rise in blood pressure in men, but not in women. After adjustments for several potential confounders, the authors could not identify a clear-cut explanation for gender differences. Their conclusion, based on the paper by Lundberg [10], is that the differences between men and women as to the response to job strain may be more related to differences in masculine and feminine social roles than to biological differences. Actually, women’s non-paid work, which is known to influence health [11], has rarely been discussed in the literature on work stress and health. 

Considering the literature on this topic, several aspects call for more research on job strain and blood pressure with female groups, such as the small numbers of studies among women [8], less consistency of data on female samples [3], weaker associations in women as compared to results among men [8], and scarcity of studies on stress that take domestic responsibilities into account [12].

The present study aims to assess whether job strain is associated with 24 h ambulatory blood pressure measurements considering the potential role of domestic work in a sample of female nursing professionals.

## 2. Experimental Section

### 2.1. Participants and Data Collection

This cross-sectional study was conducted at a public hospital in the municipality of Rio de Janeiro, Southeastern Brazil, in 2009. A total of 175 female nursing workers were selected by convenience sampling from 550 eligible workers. We did not exclude subjects under antihypertensive treatment; the inclusion of those subjects was considered a feasible solution to reduce the selection bias produced by healthy worker effect, as suggested by Ohlin *et al.* [5]. All participants worked during the day shift, and volunteered to complete a questionnaire and to wear an ambulatory blood pressure monitor (ABPM) for 24 h. 

The questionnaire provided comprehensive information on professional (paid) work hours, domestic work hours, and questions on health and healthy habits. Trained interviewers were responsible for recording the information from participants. Interviewers underwent a training course that covered the aims of the study with particular emphasis on the procedures of monitoring. The Demand-Control questionnaire was self-filled. Contact with the study participants occurred during the first three hours of a regular working shift, which began at 7 a.m. 

### 2.2. Blood Pressure Monitoring

Participants were invited to start the blood pressure monitoring procedures shortly after the interview. The 24 h blood pressure monitoring was conducted using a SpaceLabs ABPM (Model 90207, SpaceLabs Medical, Inc., Redmond, WA, USA).

The devices were programmed to measure the arterial blood pressure every 30 minutes during day and night [13]. Before starting measurements, the monitor was calibrated by comparing three successive systolic and diastolic readings against simultaneous auscultatory readings, taken by a trained observer with a mercury column, in which both had to be within 5 mm Hg to be adequate [14].

Participants were asked to perform their regular activities at work and at home during the monitoring period, and not to detach the device until the next day. They were also asked to remain still and to keep their arm beside the body during cuff inflation and deflation. Subjects recorded their location (*i.e.*, work, home or sleep), position (*i.e.*, standing, sitting or reclining) and physical activity during waking hours at the end of each measurement in a diary. In case of desistence the participant was instructed to switch off the device and keep it in a safe place.

The diary information was used to calculate the average of BP measurements while the subject was (i) at work, (ii) at home and awake and (iii) at home sleeping. When fewer than five readings were obtained for each location, the corresponding average was treated as missing data. This procedure follows the recommendations of Schnall *et al.* [15] and Llabré *et al.* [16].

### 2.3. Job Strain

Perceived stress at work was measured by the Swedish version of the Demand-Control Questionnaire [17] translated and adapted to Brazilian Portuguese by Alves * et al.* [18]. Psychological job demands were measured by five items and job control or decision latitude by six items. Each question had four frequency response categories ranging from “never” to “almost always”. 

Job strain was computed through the quadrant approach by dichotomizing decision latitude and demand scores at the medians of the sample. Subjects who presented high demand and low decision latitude scores were classified into the high strain group. The reference (low strain) group was formed by individuals who reported low demand and high decision latitude. 

Cronbach alpha values were 0.70 for psychological demands and 0.54 for decision latitude for this sample. Further analysis showed that the removal of the item which refers to repetitive tasks (*Do you have to do the same thing over and over again?*) increased the alpha from 0.54 to 0.56 for decision latitude. In addition, results from previous studies showed low psychometric performance of this item for nursing teams [19] and hospital workers [20]. Thus, we exclude the item from the statistical analyses.

### 2.4. Domestic Overload

Domestic overload considers the number of potential beneficiaries of domestic work and the person’s level of responsibility in relation to four basic household tasks: cleaning, washing, cooking and ironing [21,22]. It is defined by the total sum of scores related to each task multiplied by the number of potential beneficiaries (people who live in the house). The higher the level of responsibility for each task and/or the number of beneficiaries, the higher the domestic overload. These values were dichotomized at the second tertile of the sample distribution [23]. Subjects with a score above this value were classified as exposed to domestic overload.

### 2.5. Co-variables

Relevant covariates associated with health and with work were considered in the present study. Leisure-time physical activity was measured by the number of hours per week. Body mass index was defined by self-reported weight (kg)/height (m^2^). Smokers were defined as subjects who currently smoke or have smoked in the past. Alcohol use was measured by the consumption of alcohol drinks taken two weeks before the interview. Users of anti-hypertensive medication were those who reported making daily use of medication prescribed by a doctor. Coffee intake was defined by the median value of the sample distribution (≥250 mL/day). Oral contraceptive use was defined according to current usage. Work-related variables included extended professional work hours defined by the median value of the sample distribution (≥25 h/week) obtained by recall information corresponding to the week before the interview, professional category (nurses or nurses’ aides), social support at work, night work in the past and type of employment contract (permanent or temporary). Socio-demographic variables also included age, marital status, presence of children up to 14 years-old, family earnings and self-reported skin color.

### 2.6. Statistical Analysis

The potentially confounding effect of all variables previously described was tested using analysis of variance (ANOVA) and the Pearson’s chi-square test. Only variables that were significantly associated with BP and also with job strain were included in multivariate models. Since these data do not present a normal distribution, natural logarithm (ln) transformations were performed for mean blood pressure throughout 24 h, at work, at home and during sleep. The multivariate analyses of variance (MANOVA) were used to test the association between systolic and diastolic ambulatory BP and job strain and also other risk factors for BP alteration. Analyses were carried out for the whole 24h-monitoring period and for the periods at work, at home, and during sleep. The multivariate model included adjustment for age, self-reported skin color, and use of anti-hypertensive medication. 

Additionally, the possible interaction between job strain and domestic overload in relation to the ambulatory BP was previously assessed by the general linear model. Based on that, statistical analyses were performed for the whole sample and also stratified by domestic overload. All analyses were conducted using SPSS v.19.0 (Statistical Package for the Social Sciences, IBM).

## 3. Results

### 3.1. Sample Size and Characteristics

The sample included 175 people. However, the estimation of the mean blood pressure for some participants was not possible because there were no valid readings for some locations. The mean blood pressure at work could not be calculated for one participant. Similarly, the mean BP at home and during sleep could not be calculated for 15 and 20 participants, respectively. Thus, statistical analyses were performed for 174 participants using work ABP, for 160 participants using home ABP and for 155 participants using sleep ABP. 

The mean age was 46.1 years (standard deviation ± 11.9 years). A majority were married (53.7%), 40.8% reported being white and 59.2% reported being black or having a mixed background. With respect to family income, 51.4% of workers reported receiving more than USD 1,230 per month. Participants dedicated, on average, 20h/week (SD ± 15.4 h) to domestic work and 25.7 h/week (SD ± 12.9 h) to professional work. Among the 175 workers, 51 (29.1%) were nurses and 124 (70.9%) were nursing aides’. Most participants had only one job (67.4%) and were former night workers (72.4%). Only 23.4% practiced at least one hour of physical activity during the week, and 63.4% were overweight or obese. The sample was comprised of 32.4% of workers in active (high demand-high control) jobs, 30% in passive (low demand-low control) jobs, 22.9% in low strain (low demand-high control) jobs and 14.7% in high strain (high demand-low control) jobs.

Non-white women, anti-hypertensive medication users and workers with domestic overload had higher systolic and diastolic blood pressure at work than their counterparts. Systolic blood pressure was also higher in women over 46 years compared to younger workers (Table 1). 

**Table 1:** Crude and adjusted means and respective standard errors (SE) for ambulatory blood pressure at work according to socio-demographic and professional sample characteristics. Statistical analysis based on the multivariate analyses of variance.

Characteristics	N	Work SBP (mm Hg)		Work DBP (mm Hg)	
		Crude means (SE)	Adjusted * means (SE)	Crude means (SE)	Adjusted * means (SE)
**Age**					
≥46 years-old	93	121.1 (1.4)	122.8 (1.5)	78.4 (0.9)	79.2 (1.1)
47 or more	81	128.9 (1.5)	128.3 (1.7)	80.7 (1.0)	81.5 (1.2)
***p*****-value**		**<0.001**	**0.022**	**0.094**	0.191
**Family income (USD)**					
1,230,00 or more	95	125.4 (1.4)	125.8 (1.5)	79.8 (0.9)	80.2 (1.1)
≤1,230,00	79	123.9 (1.6)	124.6 (1.7)	79.0 (1.0)	80.3 (1.2)
***p*****-value**		0.486	0.591	0.571	0.960
Skin color **					
White	53	121.9 (1.2)	122.3 (1.7)	77.8 (0.9)	78.0 (1.2)
Non-white	77	127.5 (1.3)	127.3 (1.4)	82.0 (1.0)	81.3 (1.0)
***p*****-value**		**0.025**	**0.023**	**0.011**	**0.012**
**BMI**					
Adequate	64	119.9 (1.7)	122.8 (1.9)	77.8 (0.9)	79.5 (1.3)
Overweight/obese	110	127.6 (1.3)	126.7 (1.4)	82.0 (0.9)	80.7 (0.9)
***p*****-value**		**<0.001**	0.105	**0.074**	0.464
**Smoking**					
Never smoker	119	124.3 (1.3)	125.0 (1.3)	79.0 (1.1)	19.5 (1.3)
Smoker/former smoker	55	125.7 (1.9)	126.7 (1.4)	80.4 (0.9)	80.7 (0.9)
***p*****-value**		0.522	0.687	0.874	0.394
**Anti-hypertensive medication**					
Non treated	125	121.8 (1.2)	125.0 (1.4)	78.3 (0.8)	78.9 (0.9)
Treated	49	132.2 (1.9)	132.4 (2.4)	82.4 (1.3)	83.8 (1.6)
***p*-value**		<0.001	**0.001**	**0.007**	**0.014**
**Physical activity **					
Yes	41	123.8 (1.2)	124.8 (2.4)	80.7 (1.4)	82.7 (1.6)
No	133	125.0 (1.2)	125.4 (1.3)	79.1 (0.8)	79.6 (0.8)
***p*-value**		0.608	0.839	0.310	0.087
**Domestic overload**					
No	109	122.2 (1.2)	123.2 (1.3)	77.8 (0.8)	78.5 (0.9)
Yes	62	128.3 (1.6)	127.7 (1.7)	81.9 (1.0)	82.7 (1.2)
***p*****-value**		**0.003**	**0.036**	**0.003**	**0.007**
**Professional work hours**					
Up to 24 h/week	116	124.4 (1.3)	124.4 (1.3)	78.7 (0.8)	79.3 (0.9)
25 h or more	58	125.4 (1.8)	127.0 (2.0)	80.9 (1.2)	82.3 (1.4)
***p*****-value**		0.658	0.303	0.134	0.075
**Exposure to night work**					
Never	34	124.3 (2.0)	125.0 (2.2)	77.9 (1.3)	79.3 (1.5)
Former night worker	95	125.0 (1.2)	125.4 (1.3)	80.0 (0.8)	80.6 (0.9)
***p*****-value**		0.763	0.879	0.152	0.458

* adjusted for age, use of anti-hypertensive medication and self-reported skin color.

### 3.2. Job Strain, Ambulatory Blood Pressure, and Domestic Work

No significant associations were found between high (*vs.* low) job stain and blood pressure (Table 2). However, stratified analysis revealed that women exposed to domestic workload had a substantial association between high (*vs.* low) job strain and home systolic blood pressure (21.5 mm Hg, *p* = 0.025). Borderline significant associations for high (*vs.* low) job strain in the group with domestic overload were also observed for sleep systolic BP (16.9 mm Hg, *p* = 0.09), 24 h diastolic BP (12.9 mm Hg, *p* = 0.055), home diastolic BP (13.6 mm Hg, *p* = 0.07) and sleep systolic BP (20.5 mm Hg, *p* = 0.082).These findings suggest the existence of interaction between domestic overload and stress at work since much smaller (and non-significant) differences in mean BP due to high job strain were observed for workers with low domestic workload. The p-values of the interaction terms were <0.10 for 24 h and at home and >0.10 for work and sleep. Both active and passive groups were compared with the low strain group. No significant association was detected overall and in the two domestic overload groups

**Table 2:** Adjusted * associations between high job strain and ambulatory blood pressure (mm Hg), based on the multivariate analyses of variance.

	Whole group			Domestic Overload			No Domestic Overload		
Ambulatory Blood Pressure	High Strain (n = 25)	Low Strain (n = 39)		High Strain (n = 8)	Low Strain (n = 13)		High Strain (n = 17)	Low Strain (n = 26)	
	Mean (SE)	Mean (SE)	*p*-value	Mean (SE)	Mean (SE)	*p*-value	Mean (SE)	Mean (SE)	*p*-value
**Systolic BP**									
24 h BP	124.2 (2.9)	119.3 (2.3)	0.177	137.0 (10.8)	121.0 (7.6)	0.116	119.6 (2.5)	118.5(2.1)	0.709
At work	127.6 (2.6)	124.2 (2.3)	0.319	136.7 (7.4)	125.4 (6.2)	0.290	124.6 (2.7)	123.3(2.3)	0.710
At home	127.5 (2.6)	121.5 (2.2)	0.089	138.5 (5.7)	117.0 (4,8)	0.025	123.1 (2.7)	122.9(2.3)	0.920
During sleep	116.6 (3.6)	109.9 (2.8)	0.113	136.7 (11.4)	119.8 (7.9)	0.092	110.8 (3,1)	108.9(2.5)	0.600
**Diastolic BP**									
24 h BP	75.4 (2.3)	75.1 (1.7)	0.943	88.5 (6.7)	75.6 (4.7)	0.055	71.0 (2.1)	74.6 (1.7)	0.249
At work	80.3 (2.0)	80.5 (1.7)	0.982	89.7 (3.8)	81.5 (3.2)	0.161	76.9 (2.3)	79.6 (1.9)	0.429
At home	78.0 (2.1)	77.3 (1.8)	0.730	88.5 (4.7)	74.9 (3.9)	0.070	73.8 (2.2)	77.7 (1.8)	0.266
During sleep	68.9 (2.6)	66.6 (2.0)	0.481	86.6 (7.6)	66.1 (5.2)	0.082	63.8 (2.1)	66.0 (1.7)	0.460

* Adjusted for age, use of anti-hypertensive medication and self-reported skin color.

## 4. Discussion

The absence of association between job strain and blood pressure in the overall sample of workers is not consistent with the hypothesis that job strain would be associated with blood pressure levels in female nursing professionals. Nevertheless, the fact that significant associations were restricted to those workers subject simultaneously to job strain and to domestic overload reveals the influence of these domestic activities in the association investigated.

In the present study, the higher systolic blood pressure at home in the group exposed to job strain and to domestic overload is consistent with results by Brisson *et al.* [12], who also measured ambulatory blood pressure—in this case in women who worked in offices in Canada. The authors concluded that combined exposure to job strain and high family demands, represented by domestic responsibilities and having children, resulted in a stronger association on blood pressure than being exposed to one of these factors separately. Similar results were obtained by Aquino [24] when analyzing arterial hypertension through casual BP measurements in nursing professionals. The author observed a higher probability of hypertension among workers subject to the combination of domestic overload and accelerated work rhythm and pressure from management.

The effect of the combination of professional and domestic factors on blood pressure can be explained by the study of Lundberg and Frankenhaeuser [25] with a sample of managers (white-collar). While among men levels of noradrenalin declined after the end of a paid work day, in women the hormone levels were kept high after paid work, in the domestic environment. Considering the role of catecholamines in cardiovascular physiology, these results give biologic plausibility to the literature relating to the combination of domestic and professional spheres of life and blood pressure. 

Similarly, Goldstein and colleagues [26] observed that increase of cardiovascular activity (evaluated by cortisol secretion) due to professional work is modified by factors linked to the domestic sphere. In fact, analysis of neuroendocrine responses to stress help build an explanatory frame to the interaction observed in the present study and in other literature, since hormonal responses to stress are important to body protection and survival [10], however, in the long term, they can trigger pathologic alterations [27]. 

The influence of domestic work may explain partially the inconsistencies in the literature on job strain and blood pressure. Considering only studies based on monitoring of blood pressure in female nurses, Brown *et al.* [28] observed a significant association between job strain and blood pressure, while Riese *et al.* [29] and Goldstein *et al.* [26] did not observe such an association. (It is also possible that restriction of the sample to a single occupation in these studies led to weaker associations.) In fact, more than twenty years ago, Light *et al.* [30] pointed to domestic environment as a factor possibly associated with inconsistencies on this topic, since generally only the paid work environment is analyzed. 

It should be highlighted that unhealthy effects of simultaneous exposure to job strain and domestic responsibilities are not limited to blood pressure. The study of Ertel *et al.* [31], with a predominantly female sample, reveals a higher chance of manifestation of depressive symptoms in the group subject to job strain and family demands related to the presence of children. Krantz and Östergren [32] investigated the occurrence of physical and mental symptoms in women, having observed that both a high level of domestic responsibilities and job strain are significantly associated with a higher frequency of symptoms. The authors highlighted the synergic character of these factors—professional and domestic—since the association was even higher among women exposed simultaneously to both factors. Results presented by our research group show how much the domestic sphere is important, for example, in relation to recovery after work [33,34] and to the work ability index [35]. In fact, the need for recovery from work was shown to be more strongly associated to domestic work than to other known harmful factors, such as night shifts and effort-reward imbalance [36].

The evidence of interaction between domestic overload and job strain supports Niedhammer’s *et al.* [7] and Messing’s *et al.* [37] criticism of the use of “sex” as a confounder in studies on mixed samples in occupational health. Among the arguments used by Niedhammer *et al.* [7] is the need to consider differences between men and women in occupational exposures, prevalence of illnesses and symptoms, perception of health, responses to exposure related to biological differences, and external factors, such as activities outside of the work environment. To Niedhammer *et al.* [7], to consider sex as a confounder can lead to an overestimation or masking of the effects, hindering evaluation of occupational risk. Besides, it may lead to unreliable results if there is interaction, *i.e.*, if the association between occupational exposure and health outcome differs between males and females in direction or intensity. 

Among the limits of the present study is the cross-sectional design that does not allow us to assess causality. In addition, a number of sources of bias towards the null value may have led the observed results to be weaker than otherwise: low scale reliability for the job decision latitude component of job strain; a large proportion of the sample working less than 25 h per week in paid work (and a resulting smaller effect of paid work on BP); and limited objective variance in job characteristics (the sample being limited to two hospital job titles). Also, the relatively small sample limited statistical power for observing associations, especially in subgroups. However, despite such biases and limited power, substantial associations were observed in the group exposed to both domestic overload and job strain. 

## 5. Conclusions

Workplace psychosocial stress was not significantly associated with ambulatory blood pressure in the whole group of workers. Only women exposed simultaneously to job strain and to domestic overload showed a significantly and substantially higher level of systolic blood pressure, suggesting an important interaction of domestic work and job strain. This result occurred despite the relatively small sample size, sample composition which included female workers taking anti-hypertensive medicine, and other study limitations.

Evidence of the role of domestic work in the relationship between occupational stress and blood pressure levels may explain in part the greater inconsistency in results of studies with female samples when compared to those with male samples. The results of the present study encourage researchers to examine both the process of professional work and the domestic-family sphere when investigating female workers’ health. In sum, a gender approach is essential when considering health risks. It can be concluded that a fairer sharing of domestic responsibility and also the decrease of strain at work tend to improve the health status, thus preventing morbidity related to hypertension.
